# Three Years’ Experience of Water Purification by Means of Iron

**Published:** 1890-05

**Authors:** 


### BOOK NOTICES AND REVIEWS.

THREE YEARS' EXPERIENCE OF WATER PURIFICATION BY MEANS OF IRON, IN ANDERSON'S REVOLVING IRON PURIFIER. By E. Devonshire, Assoc. M. Inst. C. E., 3 Whitehall Place, London S. W., 1888.

This pamphlet of seventy pages and five large plates must, when its title is considered, at once arrest the attention of every practitioner of medicine.

In the July number of this journal, at page 310, we called attention to an essay by Dr. Currier, discussing the filter question. It will be recollected that he condemned all small filters, and gave a description of one at Berlin, the only one successful on the large scale.

In the pamphlet before us is a description of a most novel, yet simple, and altogether surprising method of purifying water for drinking purposes. It is nothing more or less than agitating the water with metallic iron.

We quote from the pamphlet:

"The principle of Anderson's Revolving Iron Purifier consists in the production of an intimate contact between metallic iron and the water to be purified, by the showering down of finely divided particles of the metal through an onward flowing stream of water."

To accomplish this result in a practical manner, the inventor provides a long iron cylinder of considerable diameter, that is supported horizontally upon two hollow axles. "Within the cylinder is a series of short, curved shelves, arranged in steps at equal distances round its circumference, and reaching from end to end." Into this cylinder iron in a moderately fine state of division is introduced, and spread along the bottom in sufficient quantity to occupy one-tenth of its capacity. Water is allowed to flow into the cylinder through the hollow axle, or trunnion, at one end, and permitted to escape through the other. A small engine causes the cylinder to revolve slowly, and the steps scoop up portions of the iron particles, and carry them up to the highest point of rotation, when they fall down, and, descending through the water, are arrested at the lowest point, there to be once more lifted up and allowed to drop. This process is going on continuously. The iron falling through the water purifies it, removing color, destroying bacteria and the albuminous ammonia upon which they subsist, and causing it to become limpid, even brilliant, colorless, and free from taste or smell. This result may be attained with any ordinary water "which the slowest practicable filtration through sand is powerless to render clear and bright." Portions of iron are, of course, oxidized and dissolved in the water, giving it a strong metallic taste. This is gotten rid of by exposing the water to the air, in order that the dissolved iron may oxidize. The plan for accomplishing this end is by directing it through shallow troughs to filter beds of sand, where the water stands three or four feet deep, so that it may take six hours before it reaches the sand. The sand arrests the sesquioxide of iron thus formed, and the water finally enters the reservoir, as before stated, limpid and pure.

If the water be very highly colored, as with peat, it needs a great deal of aeration to completely abolish color and opalescence.

To accomplish this, air may be pumped into the water and the water permitted to fall in cascades down a series of steps built of coke.

Air is carefully excluded from the revolving cylinder, however, and the current of water flowing through kept uniform. The iron particles under these circumstances do not rust, the continual attrition keeping their surfaces bright and smooth, and the cylinder itself entirely free from rust and scale. The best form of iron is the scrap-iron called "burrs" coming from punching machines.

The chemical theory of the process appears to be "one of reduction, to be followed, on the water leaving the apparatus, by one of oxidation." The carbonic acid contained in the impure water acts upon the metal, forming carbonate of iron. On being exposed to the air, the ferrous salt is oxidized and converted into the insoluble ferric oxide ($Fe_2 O_3$), the carbonic acid being liberated and escaping in the form of gas. From this it will be seen that proper *aeration after purification* is indispensable to complete the process. It is found that in most cases four hours' exposure to air is ample to completely precipitate all dissolved iron, which is then easily removed from the water by rapid mechanical filtration, no trace of iron remaining in the purified and filtered water.

The process has been brilliantly successful with the water of the Nile, which is charged with mud in so fine a state of subdivision that it defies any practical method of filtration.

The three years' experience with this process were obtained by practice upon the impure and highly colored waters of the river Nethe, which supplies the city of Antwerp.

The author of the book under notice is the resident engineer and manager of the Antwerp Water-works Company, limited.

He is at present traveling through the United States, endeavoring to introduce his system into the large cities. March 19th, he delivered an interesting lecture upon the subject in the Franklin Institute of Philadelphia.

The reflecting physician will find much food for thought upon the effect which this process of purifying water has upon health. W. M. J.

